# Validation of reference genes for quantitative real-time PCR studies in the dentate gyrus after experimental febrile seizures

**DOI:** 10.1186/1756-0500-5-685

**Published:** 2012-12-13

**Authors:** Ann Swijsen, Katherine Nelissen, Daniel Janssen, Jean-Michel Rigo, Govert Hoogland

**Affiliations:** 1 BIOMED Research Institute, Hasselt University and transnational University Limburg, Agoralaan Bld C, 3590, Diepenbeek, Belgium; 2 Department of Neurosurgery, school of Mental Health and Neurosciences, University Medical Center Maastricht, Maastricht, Netherlands

**Keywords:** Reference gene, Quantitative real-time PCR, Febrile seizures, Dentate gyrus

## Abstract

**Background:**

Quantitative real-time PCR (qPCR) is a commonly used technique to quantify gene expression levels. Validated normalization is essential to obtain reliable qPCR data. In that context, normalizing to multiple reference genes has become the most popular method. However, expression of reference genes may vary per tissue type, developmental stage and in response to experimental treatment. It is therefore imperative to determine stable reference genes for a specific sample set and experimental model. The present study was designed to validate potential reference genes in hippocampal tissue from rats that had experienced early-life febrile seizures (FS). To this end, we applied an established model in which FS were evoked by exposing 10-day old rat pups to heated air. One week later, we determined the expression stability of seven frequently used reference genes in the hippocampal dentate gyrus.

**Results:**

Gene expression stability of *18S rRNA*, *ActB*, *GusB*, *Arbp*, *Tbp*, *CycA* and *Rpl13A* was tested using geNorm and Normfinder software. The ranking order of reference genes proposed by geNorm was not identical to that suggested by Normfinder. However, both algorithms indicated *CycA*, *Rpl13A* and *Tbp* as the most stable genes, whereas *18S rRNA* and *ActB* were found to be the least stably expressed genes.

**Conclusions:**

Our data demonstrate that the geometric averaging of at least *CycA*, *Rpl13A* and *Tbp* allows reliable interpretation of gene expression data in this experimental set-up. The results also show that *ActB* and *18S rRNA* are not suited as reference genes in this model.

## Background

Febrile seizures (FS) are convulsions associated with fever and occur in 4% of children between the age of 3 months and 5 years [1,2]. Retrospective studies demonstrate that adult patients with hippocampal sclerosis-associated temporal lobe epilepsy (TLE) have a 40% incidence of FS, suggesting a causal relationship [2]. Also, experimental FS have a long-lasting effect on hippocampal excitability, resulting in enhanced seizure susceptibility [3-5]. At a cellular level, an altered seizure threshold may come from a change in the expression of proteins that are known to control neuronal excitability (for review see [6]). Hence, quantification of the post-FS expression of genes that encode receptors, ion channels, etc. might help elucidating FS-induced epileptogenesis.

Nowadays, quantitative real-time PCR (qPCR) is a commonly used tool to quantify gene expression. An advantage of this highly specific and sensitive technique is that it allows analysis on small amounts of starting material [7,8]. However, measured gene expression levels may be confounded by several variables during the multistep procedure of isolating and processing RNA e.g. the amount and quality of starting material, enzymatic efficiency and variability between tissues or cells in overall transcriptional activity [9-11]. Internal reference genes are most frequently used to normalize methodology-induced variations in qPCR studies [9,12]. Until recently, ‘housekeeping genes’ (HKGs) such as glyceraldehyde-3-phosphate dehydrogenase (*GAPDH*), 18S subunit ribosomal RNA (*18S rRNA*) or beta-actin (*ActB*) were commonly used as reference genes, also in neurobiological studies. HKGs are continually read and encode for products that are necessary for the metabolism and existence of a cell. They are supposed to be invariably expressed under different experimental conditions. However, the expression of these classical reference genes may vary per tissue type and developmental stage, and may even vary in response to experimental treatment [13-17]. Hence, validation of suitable reference genes for a specific sample set and experimental model is imperative to obtain consistent gene expression data. In addition, it has been shown that the accuracy of qPCR data further improves when at least three reference genes are used for normalization [9].

Although recent studies have validated reference genes for rat brain tissue in different experimental conditions [18-21], to our knowledge there is thus far no report of validated reference genes in hippocampal tissue from rats that have been exposed to experimental FS. Therefore, we used an established model where FS are evoked by exposing 10-day old rat pups to heated air [22]. One week later, we evaluated the expression stability of seven frequently used reference genes in the hippocampal dentate gyrus (DG). To this end, we used two different mathematical algorithms (geNorm [9] and Normfinder [23] VBA applets for Microsoft Excel) for normalization.

## Results

Seven reference genes (*ActB*, beta-actin; *CycA*, CyclophylinA; *18S rRNA*, 18S subunit ribosomal RNA; *Rpl13A*, Ribosomal protein L13A; *Tbp*, TATA box binding protein; *GusB*, beta-glucuronidase; *Arbp*, Acidic ribosomal phosphoprotein P0) were chosen from literature [18,24] and evaluated for gene expression stability in DG samples from 9 controls, 6 FS- and 7 FS+ rats. Despite its widespread use, *GAPDH* was not included as a candidate reference gene since other authors showed it to be unstable using the same algorithms applied in the current study [18,20]. To avoid coregulation, reference genes were selected from different functional classes. PCR efficiency of the reference genes was situated between 99.96% and 113.89% (Table 1).

**Table 1:** **Selected reference genes for analysis of expression stability**

**Gene symbol**	**Gene function**	**Primer sequence (5’ → 3’)**^**a **^**or SABiosciences qPCR assay ID**^**b**^	**Amplicon length (bp)**
*ActB*	Cytoskeletal structural protein	F: TGT CAC CAA CTG GGA CGA TA	165
		R: GGG GTG TTG AAG GTC TCA AA	
*CycA*	Serine-threonine phosphatase inhibitor	F: TAT CTG CAC TGC CAA GAC TGA GTG	126
		R: CTT CTT GCT GGT CTT GCC ATT CC	
*18S rRNA*	Ribosomal subunit	F: ACG GAC CAG AGC GAA AGC AT	310
		R: TGT CAA TCC TGT CCG TGT CC	
*Rpl13A*	Structural component of 60S ribosomal subunit	F: GGA TCC CTC CAC CCT ATG ACA	132
		R: CTG GTA CTT CCA CCC GAC CTC	
*Tbp*	General transcription factor	F: TGG GAT TGT ACC ACA GCT CCA	131
		R: CTC ATG ATG ACT GCA GCA AAC C	
*GusB*	Exoglycosidase in lysosomes	PPR43194B^b^	137
*Arbp*	Catalysis of protein synthesis	PPR42394A^b^	92
*Cnr1*	Endocannabinoid signalling	PPR52793A^b^	156

*ActB*, beta-actin; *CycA*, CyclophilinA; *18S rRNA*, 18S subunit ribosomal RNA; *Rpl13A*, Ribosomal protein L13A; *Tbp*, TATA box binding protein; *GusB*, beta-glucuronidase; *Arbp*, Acidic ribosomal phosphoprotein P0; *Cnr1*, Cannabinoid Type 1 (CB1) receptor. F, forward primer; R, reverse primer.

^a^ primer sequences are based on literature [24].

^b^ ID, identification.

### Cycle threshold values of candidate reference genes

**Figure 1:**
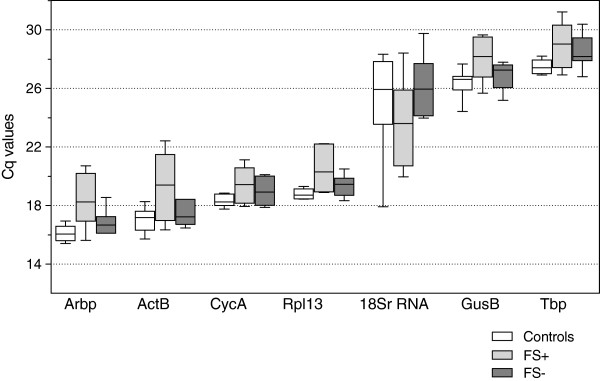
**Cycle threshold (Cq) levels of candidate reference genes in each experimental group.** Boxes represent lower and upper quartiles with medians, whiskers represent the outer 10%. Normothermia controls (n = 9); FS-, hyperthermia without febrile seizures (n = 6); FS+, hyperthermia with febrile seizures (n = 7).

When all samples were taken together, the reference genes showed cycle threshold (Cq) values varying from a Cq value of 15.40 for *Arbp* to 31.22 for *Tbp* (Additional file 1). With a Cq ≤ 22.23, *Arbp*, *ActB*, *CycA* and *Rpl13A* showed lower Cq values than *GusB* and *Tbp* that had a Cq ≥ 24.42. *18S rRNA* displayed the highest Cq variability, ranging from 17.91 to 29.75. A similar Cq pattern was observed when Cq values were calculated per experimental group (Figure 1). Cq standard deviations provide a first idea about the variability in expression, ranking from most to least variably expressed as *18S rRNA* > *ActB* > *Arbp* > *GusB* > *Tbp* > *Rpl13A* > *CycA*.

### Validating candidate reference genes

**Figure 2:**
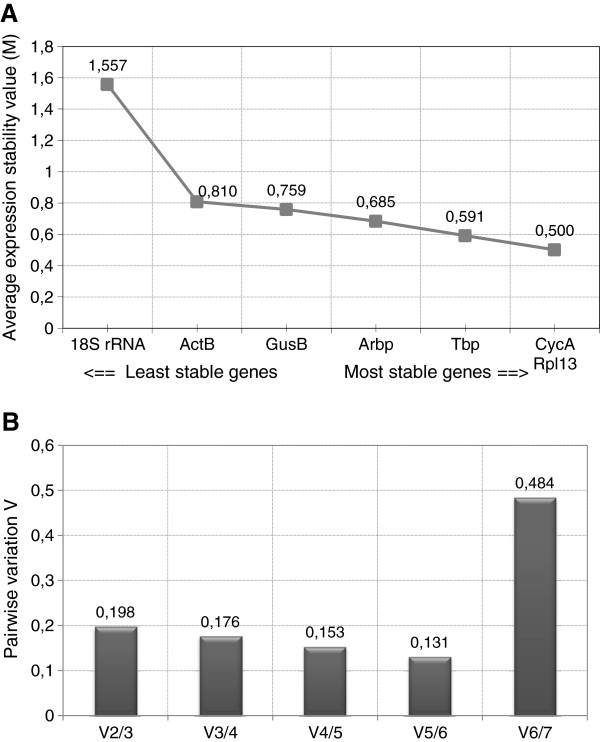
**Evaluation of candidate reference genes using geNorm analysis software. A**: Average expression stability measure (M) of reference genes in the total sample set (n = 22), analyzed by stepwise exclusion of the least stable reference gene. **B**: Determination of the optimal number of reference genes for normalization by means of pair-wise variation (V_n/n+1_) analysis. Every bar represents the pairwise variation (V) in normalization accuracy when stepwise adding reference genes according to the ranking in **A**.

geNorm is used to determine the average expression stability (M value), based on the average pairwise variation between a particular gene and all other reference genes in the study. With the exception of *18S rRNA*, all genes did show high expression stability, indicated by M values below the default limit of 1.5 suggested by the geNorm software (Figure 2A). Based on the M values, *CycA* and *Rpl13A* were the most stably expressed genes. It is commonly known that normalization to multiple reference genes is advisable, as the use of a single gene may introduce normalization errors. In that respect, geometric averaging of multiple reference genes is a proven method to calculate an accurate normalization factor [9]. The optimal number of reference genes to be used for normalization can be determined by pairwise variation between two sequential normalization factors (NF_n_ and NF_n+1_), starting with genes with the lowest M value. This analysis learned that for this dataset, the use of five reference genes is optimal when a variation value (V_n/n+1_) < 0.15 is considered as not significantly improving the accuracy (Figure 2B). However, inclusion of the fourth (V_3/4_ = 0.176) and fifth (V_4/5_ = 0.153) most stable gene causes only slight differences in the pairwise variation value (Figure 2B).

**Figure 3:**
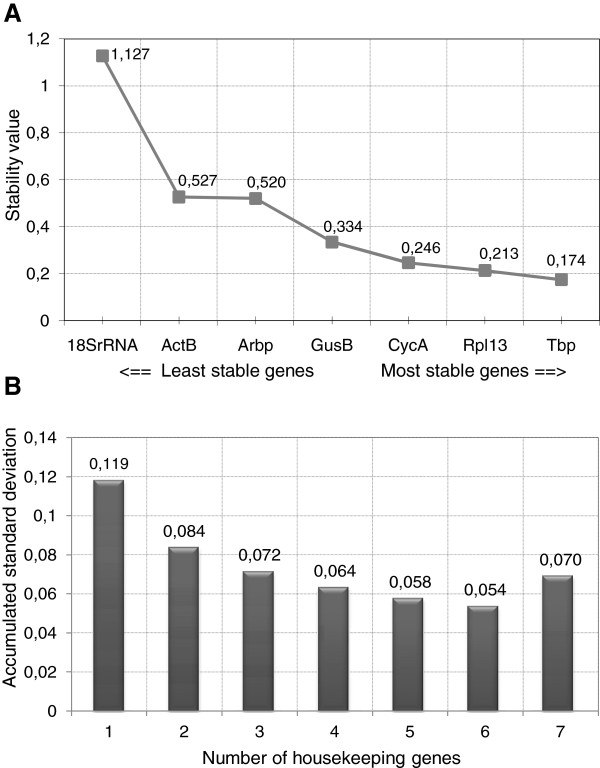
**Evaluation of candidate reference genes using Normfinder analysis software. A**: Stability values of each reference gene in the total sample set (n = 22). **B**: Determination of the optimal number of reference genes for normalization using the accumulated standard deviation (Acc. SD).

The stability ranking of the candidate reference genes determined by geNorm was compared with Microsoft Excel-based applet termed Normfinder. This algorithm provides a stability value for each candidate reference gene and ranks the genes according to their expression stability in a given sample set and experimental design. Normfinder also identified *Rpl13A* as one of the most stably expressed genes, and *18S rRNA* as the least stable gene (Figure 3A). Yet, *Tbp* was identified as the most stably expressed gene. This algorithm enables to calculate an Acc.SD, which is an indicator of the optimal number of reference genes necessary to obtain an accurate normalization factor. By this approach, we found that an Acc.SD of 0.119 using one gene could be lowered to 0.054 when six genes were included (Figure 3B). Most of this decrease in Acc.SD was attributable to the first three genes (Acc.SD = 0.072).

The ranking order of reference genes proposed by geNorm was not identical to that suggested by Normfinder. However, both algorithms indicated *CycA*, *Rpl13A* and *Tbp* as the most stable genes, whereas *18S rRNA* and *ActB* were found to be the least stably expressed genes (Table 2).

**Table 2:** **Ranking of reference genes based on the expression stability evaluated by geNorm and Normfinder**

**Ranking order**	**geNorm**	**Normfinder**
**1**	*CycA* – *Rpl13A*	*Tbp*
**2**		*Rpl13A*
**3**	*Tbp*	*CycA*
**4**	*Arbp*	*GusB*
**5**	*GusB*	*Arbp*
**6**	*ActB*	*ActB*
**7**	*18S rRNA*	*18S rRNA*

### Influence of different normalization approaches on the expression profile of a gene of interest

**Figure 4:**
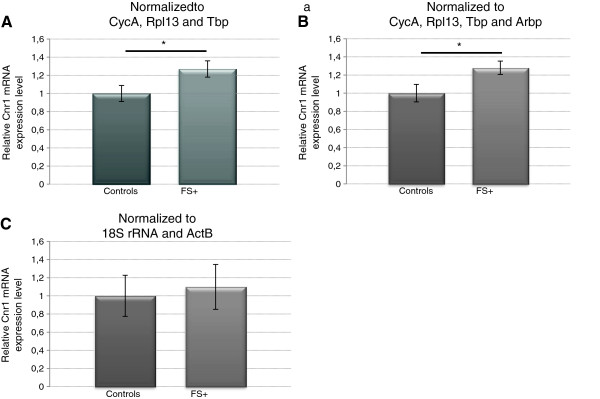
**Influence of reference genes selected for normalization on the expression profile of *Cnr1 *one week after FS induction.***Cnr1* expression levels were normalized by geometric averaging of three (**A**) or four (**B**) stably expressed genes as identified by geNorm and Normfinder. *18S rRNA* and *ActB*, indicated by geNorm and Normfinder as the least stable genes, are used for normalization of *Cnr1* expression data (**C**). Normothermia controls (n = 9); FS+, hyperthermia with febrile seizures (n = 7). Data are presented as mean ± SEM. *, P < 0,05; analyzed using a Mann–Whitney test.

To demonstrate the importance of choosing sufficient and stably expressed reference genes, we normalized the expression of the cannabinoid type 1 receptor gene *Cnr1* to different normalization factors. This gene of interest was chosen because it was previously shown that hippocampal protein levels of this receptor are significantly increased one week after experimental FS [25]. Here, we first normalized *Cnr1* to the geometric average of the three reference genes (*CycA*, *Rpl13A* and *Tbp*) that were indicated as most stably expressed by geNorm and Normfinder analysis. This resulted in a significant upregulation of *Cnr1* in FS + rats, compared to controls (Figure 4A). Inclusion of the fourth most stable gene suggested by geNorm, *Arbp*, in the normalization factor did not change *Cnr1* expression levels (Figure 4B). However, the significance of *Cnr1* upregulation disappeared when the signal was normalized to the two commonly used reference genes *18S rRNA* and *ActB*, which were identified as the most unstable genes by both algorithms (Figure 4C). This normalization strategy also caused a strong increase in the standard error.

## Discussion

Validated normalization is crucial to obtain reproducible qPCR data of genes of interest. In this context, normalizing to internal reference genes has become the most popular method to control for experimental errors introduced by the multitude of steps in this analysis. Several studies point out that the expression of reference genes may vary under different experimental conditions [13-17]. This implies the necessity of validating these genes in each new experimental setup.

To our knowledge, this is the first study that describes the stability of *18S rRNA*, *ActB*, *GusB*, *Arbp*, *Tbp*, *CycA* and *Rpl13A* in the DG of rats one week after FS. Both, geNorm and Normfinder were used to rank the analyzed reference genes by their expression stability. This rank order differed slightly between both methods, probably because both tools are based on different mathematical models. Other studies have also described similar ranking discrepancies between geNorm and Normfinder [18,24]. Interestingly though, both programs agreed on the three most stably expressed genes, being *CycA*, *Tbp* and *Rpl13A*. These converging results stress the significance of including these genes in the normalization factor. In addition, both programs also agreed on *ActB* and *18S rRNA* as the least stably expressed genes. Comparison of these data with those of recent studies revealed similarities and differences. For instance, Bonefeld et al. [20] validated eight reference genes in rat hippocampal tissue and also identified *CycA* and *Rpl13A* as the most stably expressed genes and *ActB* and *18S rRNA* as the least stable genes. Also Pernot et al. [18] found that *CycA* and *Tbp* were stably expressed in hippocampus samples from a mouse model of TLE, obtained across different phases of the disease. However, in contrast to our study they also observed a stable *ActB* expression. This discrepancy emphasizes the importance of validating reference genes in each experimental model.

Accurate normalization requires inclusion of multiple reference genes. Geometric averaging of the most stable reference genes is a validated method to obtain a reliable normalization factor [9]. Based on a cut-off value of 1.5, geNorm indicated that the normalization factor should be based on five reference genes. However, according to the geNorm manual, this cut-off value can be set differently. geNorm calculates the optimal number of reference genes by pairwise variation analysis. In that respect, the trend of changing V values after adding additional genes can be used to obtain an estimate of the number of genes that should be included in the normalization factor. Determination of the optimal number of reference genes is always a trade-off between accuracy and practical considerations, but a minimum of three most stable reference genes is generally recommended [9]. As indicated in Figure 2B, a pairwise variation of 0.198 was observed after adding the third most stable gene. Inclusion of the fourth or fifth most stable gene influenced only slightly the pairwise variation. The high V_6/7_ is caused by the high average M value of *18S rRNA*, indicating that this gene is highly variably expressed under the present experimental conditions. The Acc.SD calculated by Normfinder suggested the use of six reference genes, though the additive value of genes four to six is minimal. Considering the pairwise variation values, the Acc.SD, and practical issues such as the available amount of RNA, we conclude that the geometric mean of *CycA*, *Rpl13A* and *Tbp* should be used to obtain an accurate normalization factor in this experimental setup. If the RNA yield allows the inclusion of an extra reference gene, *GusB* and *Arbp* may be added to this panel. These data also show that *18S rRNA* is unfit as reference gene in this model.

Inclusion of the geNorm/Normfinder selected reference genes in the normalization factor, revealed an increased *Cnr1* expression in animals that experienced FS (FS+). This finding is in agreement with quantitative western blot data from Chen et al. [25]. This upregulated *Cnr1* disappeared when expression levels were normalized to *ActB* and *18S rRNA*, underscoring the suggestion that these ‘classical’ reference genes are not suitable for our experimental setup. In line with this observation, several studies have reported that including *18S rRNA* or *ActB* in the normalization factor altered mRNA expression levels compared to normalization to geNorm proposed genes [18,20]. As a possible explanation for erroneous normalization when *18S rRNA* is used as reference gene, it has been suggested that this may relate to an imbalance between messenger RNA and ribosomal RNA [17].

## Conclusions

In conclusion, the present study describes the expression stability of seven candidate reference genes in the hippocampal DG of rats, one week after FS. Our results demonstrate that the geometric averaging of at least *CycA*, *Rpl13A* and *Tbp* allows a reliable interpretation of mRNA expression data in this experimental set-up. These data also show that *ActB* and *18S rRNA* are unfit to serve as reference gene in this model.

## Methods

### Induction of febrile seizures and tissue sampling

Litters of 5–10 male Sprague–Dawley rat pups (Harlan, Horst, The Netherlands) were housed with a dam under temperature controlled conditions and 12 h dark–light cycle with water and food ad libitum. At postnatal day 10, FS were evoked by hyperthermia as described before [26-29]. Briefly, pups were injected subcutaneously with 0.2 ml 0.9% NaCl to prevent dehydration, placed in a perspex cylinder and exposed to a regulated stream of heated air. Rectal temperatures were monitored every 2.5 min. A core temperature >39.5°C (usually reached within 5 min) indicated the start of a 30 min hyperthermia phase in which the heated air stream was adjusted to maintain a core temperature of 41–42.5°C. Behavioral seizures occurring during treatment (FS+), were monitored by two observers. These seizures were stereotyped and previously shown to correlate with rhythmic epileptic discharges in the hippocampus [26]. Some rats did not display seizure behavior during the hyperthermia phase (FS-). The hyperthermia phase was terminated by dipping the pup in room temperature water, until the pre-treatment body temperature was reached and then returned to the dam. Normothermia control rats underwent the same treatment, except that the stream of air was adjusted to maintain the body temperature that was measured at the start of the experiment (~35°C). Six to nine days after FS induction, rats were decapitated, brains were rapidly removed from the skull and placed in ice-cold oxygenated (95%O_2_/5%CO_2_) sucrose-based artificial cerebrospinal fluid (sucrose-aCSF) containing (in mM): 210 sucrose, 2.5 KCl, 26 NaHCO_3_, 1.25 NaH_2_PO_4_, 25 glucose, 1 CaCl_2_, and 7 MgSO_4_ (pH 7.4, ~340 mOsm). Next, 350-μm-thick coronal slices were cut in ice-cold oxygenated sucrose-aCSF using a vibratome (Microm/Thermo Fisher Scientific, Walldorf, Germany) and DG regions were microdissected from each acute brain slice. DG samples were then quickly frozen in liquid nitrogen and stored at −80°C until RNA isolation. All experiments were approved by the Hasselt University ethics committee for animals.

### RNA isolation and cDNA synthesis

Total RNA was isolated from DG samples using the RNAqueous-Micro kit (Ambion, Lennik, Belgium), according to the manufacturer’s protocol. Trace amounts of genomic DNA were removed by DNase I provided with the kit. RNA purity and concentration were checked by optical density, using a NanoDrop ND-1000 spectrophotometer (Thermo Fisher Scientific, Waltham, USA). For cDNA synthesis, total RNA (220 ng) was first incubated for 10 min at 70°C in order to prevent secondary structures, and then reverse transcribed using the Reverse Transcription System (Promega, Leiden, The Netherlands) in a 20 μl reaction volume containing 5 mM MgCl_2_, 1× Reverse Transcription buffer, 1 mM dNTP mixture, 0.25 μg Oligo(dT)15 primers, 0.25 μg hexamer oligonucleotides, 20 U RNase inhibitor and 12.5 U AMV reverse transcriptase, that was first incubated for 60 min at 42°C, then for 5 min at 95°C and then at 4°C. All cDNA samples were stored at −20°C until qPCR analysis.

### Quantitative real-time PCR

qPCR was performed in optical 96-well plates with an ABI PRISM 7500 Fast sequence detection system (Applied Biosystems, Carlsbad, California), and carried out in a 10 μl reaction volume containing 5 μl RT SYBR green qPCR master mix (SABiosciences/Qiagen, Venlo, The Netherlands), 0.4 μM forward and reverse primer (Table 1), and 11 ng cDNA dissolved in nuclease-free water. A no-template control containing nuclease-free water instead of cDNA was included to test for possible contamination of assay reagents. Samples were run in duplicate. PCR conditions comprised a 10 min preincubation at 95°C, followed by 40 cycles of 15 s at 95°C and 60 s at 60°C. Fluorescence was measured at 522 nm wavelength during each annealing step. Each PCR program was followed by a general dissociation curve protocol to check product specificity. PCR efficiency of the reference genes was determined by a standard curve of cDNA samples according to the MIQE guidelines [30].

### Data analysis

RNA copy numbers were quantified using the comparative ΔΔCt method as follows. Raw Cq values were first transformed to quantities. These raw reference gene quantities that are expressed relative to the sample with the highest quantity, served as data input for geNorm [9] or Normfinder [23]. The geNorm algorithm provides a measure of gene expression stability (M value) and determines the optimal number of reference genes using pairwise variation (V) analysis. In contrast to geNorm, Normfinder estimates not only the overall expression variation of the candidate reference gene, but also the variation between sample subgroups. The output of Normfinder consists of a stability value based on both intra- and intergroup expression variation. The Accumulated Standard Deviation (Acc.SD), as indicator for the optimal number of reference genes, was determined using GenEx software. For each sample, the normalization factor based on *n* reference genes was calculated as the geometric average of the *n* raw reference gene quantities.

Data are presented as mean ± standard error of the mean (SEM). Statistical analysis was performed using Graphpad Prism5 software. Differences between means were tested using the Mann–Whitney test. A value of P < 0.05 was considered as statistically significant.

## Abbreviations

18S rRNA: 18S subunit ribosomal RNA;ActB: Beta-actin;Arbp: Acidic ribosomal phosphoprotein P0;CycA: CyclophylinA;Cq: Cycle threshold;DG: Dentate gyrus;FS: Febrile seizures;GAPDH: Glyceraldehyde-3-phosphate dehydrogenase;GusB: Beta-glucuronidase;HKG: Housekeeping gene;qPCR: Quantitative real-time PCR;Rpl13A: Ribosomal protein L13A;Tbp: TATA box binding protein;TLE: Temporal Lobe Epilepsy

## Competing interests

We declare that we have no competing interests.

## Authors' contributions

AS designed and performed the experiments, analyzed and interpreted the data and wrote the manuscript. KN designed the primers and was involved in the analysis and interpretation of the data. DJ helped in generating the animal model and tissue sample collection. JMR and GH designed the research project, supervised the study, were involved in the interpretation of the data and critically revised the manuscript. All authors have read and approved the final manuscript.

## Supplementary Material

Additional file 1 **Supplementary Table – Cq values of candidate reference genes.** Table of Cq values of all candidate reference genes evaluated in the microdissected dentate gyrus in each experimental condition. Normothermia controls (n = 9); FS-, hyperthermia without febrile seizures (n = 6); FS+, hyperthermia with febrile seizures (n = 7). Click here for file
