# Supplementary material for: Validation of reference genes for quantitative real-time PCR studies in the dentate gyrus after experimental febrile seizures
Source: BMC Res Notes. 2012 Dec 13;5:685. doi: 10.1186/1756-0500-5-685 (PMC3598510; doi:10.1186/1756-0500-5-685)
Supplement: Additional file 1 — Supplementary Table – Cq values of candidate reference genes. Table of Cq values of all candidate reference genes evaluated in the microdissected dentate gyrus in each experimental condition. Normothermia controls (n = 9); FS-, hyperthermia without febrile seizures (n = 6); FS+, hyperthermia with febrile seizures (n = 7). [file 1756-0500-5-685-S1.pdf]

Additional file 1: Supplementary Table - Cq values of candidate reference genes

			Gus B	Arbp	ActB	CycA	18S rRNA	Rpl13	Tbp
**Controls**	Samples	1	26,60	16,30	17,29	18,84	24,72	19,13	27,79
		2	26,67	15,56	16,53	18,25	28,33	18,54	28,20
		3	24,42	16,94	17,41	18,81	22,38	19,14	27,41
		4	26,36	16,50	16,60	18,24	17,91	18,43	26,91
		5	26,83	16,05	17,79	18,22	27,55	19,04	28,03
		6	27,67	16,66	18,27	18,76	25,39	19,30	27,86
		7	26,80	16,02	17,17	18,17	26,02	18,71	27,07
		8	26,61	15,40	16,10	17,75	25,93	18,44	26,96
		9	25,43	15,64	15,71	17,83	28,13	18,46	27,19
**FS+**	Samples	1	27,80	17,21	17,22	19,05	26,97	19,45	28,84
		2	27,25	17,23	18,42	20,01	29,75	20,50	30,39
		3	27,60	18,55	18,42	20,11	24,13	19,86	29,46
		4	26,46	16,66	16,70	18,39	23,97	19,06	28,05
		5	26,05	16,10	16,83	18,01	27,69	18,69	27,90
		6	25,19	16,11	16,46	17,87	25,51	18,33	26,79
		7	27,43	16,65	17,72	18,92	25,95	19,64	28,18
**FS-**	Samples	1	27,15	15,62	17,18	18,23	28,41	18,95	27,59
		2	25,67	17,37	16,34	19,01	20,96	18,88	26,93
		3	28,72	17,85	18,62	19,84	22,55	20,81	29,05
		4	29,45	20,01	21,16	21,12	19,96	22,20	30,03
		5	27,62	18,64	20,17	17,93	24,64	19,77	29,02
		6	29,65	20,70	22,41	20,39	25,04	22,23	31,22
	Mean Cq		26,97	16,99	17,75	18,81	25,09	19,44	28,22
	SD		1,28	1,41	1,66	0,94	2,95	1,11	1,22
